# Perioperative Outcomes of Systematic Mesopancreas Dissection for Pancreatic and Periampullary Carcinoma at a Tertiary Referral Center From a Low Middle-Income Country

**DOI:** 10.7759/cureus.42461

**Published:** 2023-07-25

**Authors:** Sujan Shrestha, Romi Dahal, Narendra Maharjan, Bishnu Kandel, Paleswan Joshi Lakhey

**Affiliations:** 1 Department of Surgical Gastroenterology, Institute of Medicine, Tribhuvan University Teaching Hospital, Kathmandu, NPL

**Keywords:** systematic mesopancreas dissection, periampullary cancer, pancreaticoduodenectomy, pancreatic cancer, mesopancreas

## Abstract

Introduction

Systematic mesopancreas dissection (SMD) is an emerging surgical approach in pancreatic cancer surgery. There is still debate about early postoperative and pathological outcomes using SMD in pancreatic cancer surgery. This study has been conducted to compare the perioperative outcomes, the lymph node yield, and the margin status in patients who underwent standard pancreaticoduodenectomy (ST-PD) and SMD-PD for pancreatic and periampullary carcinoma.

Methods

A retrospective comparative study was conducted in patients who underwent PD for pancreatic and periampullary carcinoma in a single unit of gastrointestinal and hepatopancreatobiliary surgery at Tribhuvan University Teaching Hospital, Nepal. Early perioperative and pathological outcomes were compared between the SMD-PD and ST-PD.

Results

The demographic data of 30 patients who underwent SMD-PD was comparable with the historical data of 40 patients who underwent ST-PD. The intraoperative blood loss and postoperative complications were found to be comparable between ST-PD and SMD-PD. However, the median operative time for SMD-PD was longer than ST-PD (360 minutes [IQR: 90 minutes] vs. 360 minutes [IQR: 60 minutes]). The rate of margin negative resection was similar between both groups. The median lymph node yield was significantly high in patients who underwent SMD-PD (17.5 (IQR: 6.5) vs. 11 [IQR-10.75]; p < 0.05).

Conclusion

SMD is safe and feasible for treating periampullary carcinoma and is particularly helpful in increasing lymph node yield.

## Introduction

Pancreatic and periampullary carcinoma accounts for approximately 0.2% of all GI tract tumors, but there has been an increase in incidence in recent years [1]. Tumors of periampullary carcinoma are usually managed in a similar way but carry different prognoses [2]. Pancreatic cancer carries the worst prognosis, with overall survival of around 5% [1]. Surgery is still the only curative option available for pancreatic and periampullary carcinoma, but only 15-20% of patients are candidates for resection [3]. In periampullary carcinoma (ca), several pathological components such as tumor size and location, site of origin, degree of differentiation, lymphovascular and neural invasion, lymph node involvement, and surgical margin status are of great importance. Extended pancreaticoduodenectomy (PD) and extensive lymphadenectomies have been proposed to increase R0 resection rates and consequently optimize the oncological outcomes [4]. The presence of lymph node metastasis is associated with poor prognosis in the case of periampullary carcinomas [5]. Adequate and systematic lymph node sampling is considered a good indicator of the quality of surgical procedures and pathological handling. To standardize surgical procedures in pancreatic and periampullary cancers, the concept of mesopancreas and systematic mesopancreas dissection (SMD) was developed [6]. Mesopancreas is considered the primary site for positive resection margins, which may account for the high rate of local recurrence in resectable pancreatic head cancers [7]. Many recent studies have observed the positive impact of SMD on negative surgical margins and adequate nodal clearance in pancreatic and periampullary carcinoma [8]. With the evolution in mesopancreas-based surgical dissection in the pancreas and periampullary carcinoma, we provide our contribution to this issue. This study compared the early perioperative and pathological outcomes between SMD during SMD-PD and standard pancreaticoduodenectomy (ST-PD) in pancreatic and periampullary carcinoma.

## Materials and methods

This is a retrospective study from a prospectively maintained database conducted in the Department of Surgical Gastroenterology, Tribhuvan University Teaching Hospital, Kathmandu, Nepal. All cases of pancreatic and periampullary carcinoma operated by a single team from April 2013 to April 2021 were included. Ethical approval to conduct the study was obtained from the Institutional Review Committee of the Institute of Medicine, Tribhuvan University, with reference number 577(6-11)E2.

We started performing SMD during SMD-PD at our institution in January 2018, as described by Inoue Y et al. [9]. Thus, we divided all patients who underwent PD for pancreatic and periampullary carcinoma into two groups. Patients operated on from April 2013 to December 2017 were included in the ST-PD group, and from January 2018 to April 2021, they were included in the SMD-PD group.

The preoperative evaluation was done with a pancreatic protocol CT scan to stage our patients and assess general health and nutritional status. Preoperative biliary drainage and biopsy were advised when indicated. Procedure-specific complications such as postoperative pancreatic fistula (POPF), post-pancreatectomy hemorrhage (PPH), and delayed gastric emptying (DGE) were defined as per International Study Group of Pancreatic Surgery (ISGPS) definitions [10-12]. Postoperative morbidity and mortality were defined as per Clavien Dindo classification [13]. Patient demographics, intraoperative blood loss, operative time, postoperative complications, tumor stage, and histopathological data were collected and analyzed. The final diagnosis was based on the pathological findings.

Statistical analysis

The results were expressed as the median (range) for the quantitative data, and differences between the two groups were compared using the Mann-Whitney U test. The categorical data were compared using the χ2 and Fisher’s exact tests. P-value <0.05 was taken as statistically significant. All data were analyzed using SPSS version 23.0 (IBM Corp., Armonk, NY).

## Results

Patient demographics

Out of 70 patients who underwent PD during the study period, 30 underwent SMD-PD and 40 underwent ST-PD. The median age in SMD-PD and ST-PD were 56 and 53 years, respectively. There were 17 (56.66%) and 21 (52.5%) male patients in SMD-PD and ST-PD, respectively. Preoperative drainage was performed in 10 (33.33%) and 15 (37.5%) patients in SMD-PD and ST-PD, respectively. Among SMD-PD and ST-PD, there were no significant differences in age, gender, and rate of preoperative biliary drainage (Table 1).

**Table 1:** Demographics and clinical characteristics of patients. ST-PD: Standard pancreaticoduodenectomy; SMD-PD: Systematic mesopancreas dissection during pancreaticoduodenectomy; ca: carcinoma; ERCP: Endoscopic Retrograde Cholangiopancreatography; PTBD: Percutaneous Transhepatic Biliary Drainage.

Variables	ST-PD	Percentage (%)	SMD-PD	Percentage (%)	P-value
Sex					0.729
Male	21	52.5%	17	56.7%	
Female	19	47.5%	13	43.3%	
Diagnosis					0.956
Ampullary ca	22	55%	18	60%	
Pancreatic ca	9	22.5%	7	23.3%	
Distal cholangioca	6	15%	4	13.3%	
Duodenal ca	3	7.5%	1	3.3%	
Preoperative drainage					0.489
No	25	62.5%	20	66.7%	
ERCP	8	20%	3	10%	
PTBD	7	17.5%	7	23.3%	

Surgical outcomes

Median operative time was 360 minutes (IQR: 60 minutes) and 360 minutes (IQR: 90 minutes) for ST-PD and SMD-PD, respectively. Similarly, the median intraoperative blood loss for ST-PD was 500 milliliter (ml) (IQR: 200 ml), and for SMD-PD was 450 ml (IQR: 200 ml). Median hospital stay was 13 days (IQR: 12 days) and 14 days (IQR: 5 days), respectively, for ST-PD and SMD-PD. Major complications (Clavien-Dindo 3) were present in 32.5% and 30% of ST-PD and SMD-PD, respectively. Procedure-specific complications were similar between both groups. Hospital mortality occurred in five ST-PD cases and two SMD-PD cases (Table 2).

**Table 2:** Surgical outcomes of patients. ST-PD: Standard pancreaticoduodenectomy; SMD-PD: Systematic mesopancreas dissection during pancreaticoduodenectomy; CR-POPF: Clinical relevant postoperative pancreatic fistula; PPH: Postpancreatectomy hemorrhage; DGE: Delayed gastric emptying; SSI: Surgical site infection; CD: Clavien Dindo.

Variables	ST-PD	SMD-PD	P-value
Intraoperative blood loss (milliliter) Median (IQR)	500 (200)	450 (200)	0.834
Duration of surgery (minutes) Median (IQR)	360 (60)	360 (90)	0.039
Postoperative complications-number (%)			
CR-POPF			0.426
B	5 (12.5%)	5 (16.7%)	
C	7 (17.5%)	2 (6.7%)	
PPH	10 (25%)	3 (10%)	0.11
DGE	5 (12.5%)	2 (6.7%)	0.687
Bile leak	2 (5%)	-	0.323
SSI	15 (37.5%)	16 (53.3%)	0.187
Complications number (%)			
CD$\geq$1	28 (70%)	24 (80%)	0.343
CD$\geq$3	13 (32.5%)	9 (30%)	0.824
CD​​=5	5 (12.5%)	2 (6.7%)	0.687
Length of hospital stay (days) median (IQR)	13 (12)	14 (5)	0.759

Pathological outcomes

Out of 70 patients who underwent PD, 40 patients were operated on for ampullary carcinoma and 18, 11, and 4 patients were operated on for pancreatic ductal adenocarcinoma, distal cholangiocarcinoma, and duodenal carcinoma, respectively. The median tumor size was 2.45 cm (IQR: 2.6 cm) in ST-PD and 2 cm (IQR: 2 cm) in SMD-PD. Grade three and grade four differentiated tumors were present in four (10%) and three (10%) patients in ST-PD and SMD-PD, respectively. Margin-positive resections were present in four cases (10%) in ST-PD and three (10%) in SMD-PD. The superior mesenteric artery margin (SMA margin) was the only site of margin positive in all cases. Perineural invasion (PNI) was present in 14 patients (35%) and 16 patients (53.3%) in ST-PD and SMD-PD, respectively. Lymphovascular invasion (LVI) was present in 19 patients (47.5%) and 12 patients (40%) in ST-PD and SMD-PD, respectively. The average nodal yield was 11 (IQR: 10.75) in ST-PD and 17.5 (IQR: 6.5) in SMD-PD (Table 3).

**Table 3:** Pathological outcomes. ST-PD: Standard pancreaticoduodenectomy; SMD-PD: Systematic mesopancreas dissection during pancreaticoduodenectomy; LVI: Lymphovascular invasion; PNI: Perineural invasion; R: Resection margin.

Variables	ST-PD	SMD-PD	P-value
T stage (%)			0.944
T1-T2	23 (57.5%)	17 (56.7%)	
T3	17 (42.5%)	13 (43.3%)	
Total Nodal Yield (Median/IQR)	11 (10.75)	17.5 (6.5)	0.004
Grade of tumor (%)			0.671
G1	17 (42.5%)	9 (30%)	
G2	19 (47.5%)	18 (60%)	
G3	2 (5%)	2 (6.7%)	
G4	2 (5%)	1 (3.3%)	
LVI (%)	19 (47.5%)	12 (40%)	0.532
PNI (%)	14 (35%)	16 (53.3%)	0.125
R1 Status (%)	4 (10%)	3 (10%)	1

## Discussion

PD is a complex surgical procedure for periampullary carcinoma and pancreatic head adenocarcinoma. Mortality and morbidity associated with PD is the main factor that limits this procedure to experienced surgeons and high-volume centers. Long-term survival following PD not only depends upon the biology of the tumor but also on the quality of the surgical procedure. Early postoperative mortality, morbidity, and histological reports are the indirect tools that predict the quality of surgery. The importance and concept of mesopancreas were described in 2007 by Gockel I et al. [6]. Three levels of SMD, indication, surgical technique, and early perioperative outcomes, were described by Inoue Y et al. [9]. In this study, we have used the same levels of SMD as described by Inoue Y et al. There were two main goals for conducting this study. Firstly, we wanted to compare the histological or pathological variables between ST-PD and SMD-PD. Secondly, we wanted to evaluate different intraoperative variables and early postoperative outcomes between ST-PD and SMD-PD. In a study by Inoue Y et al., the amount of blood loss and operative duration was less with the SMD-PD when compared with their conventional PD [9]. In our study, the intraoperative blood loss was similar between the SMD-PD and ST-PD groups, contrasting the findings of Inoue Y et al. ST-PD, a complex procedure involving systematic dissection, requires sufficient experience to meet the benchmarks set by Inoue et al. This learning curve associated with ST-PD may explain the similar early postoperative outcomes between our two study groups. The total operative duration was longer for SMD-PD. All patients in the SMD-PD group had their mesentery, supplied by the first jejunal artery, removed with the specimen. The type of pancreaticojejunostomy (PJ) for all ST-PD cases and 18 cases of SMD-PD was end-to-end dunking. Modified Blumgart duct to mucosa PJ was done in 12 cases of SMD-PD. Additional jejunal mesentery resection and complex duct to mucosa PJ might be the reason for the longer operative duration for SMD-PD. Procedure-specific complications like DGE and POPF were more in the conventional PD group in the study by Inoue Y et al. [9]. Stapled gastrojejunostomy and more hard texture of the pancreas in the SMD-PD group might be the possible reason for reduced DGE and POPF in the SMD-PD group in a study by Inoue Y et al. [9]. The early postoperative complications like POPF, DGE, and PPH were similar between both groups in our study. Macroscopic (R2) or microscopic (R1) resection margins will determine the local recurrence. In most cases, the soft tissue that contains various lymphatic, nervous, and vascular structures located between the SMA and region from the pancreatic head to the uncinate process is the main site for R1 resection [14,15]. In this study, we achieved a microscopic margin negative (R0) in 90% of patients in both ST-PD and SMD-PD. Among the periampullary cancers, pancreatic ductal adenocarcinoma is associated with a few R0 resection margin statuses and is also a more aggressive cancer [16]. The possible reason for similar resection margin status between ST-PD and SMD-PD might be due to a smaller number of pancreatic ductal adenocarcinoma cases. More than half of our patients were operated for ampullary carcinoma.

One of the principal factors for loco-regional recurrence is the presence of nodal metastasis and adequacy of nodal harvest. At least 15 lymph nodes should be detected during PD to ensure adequate pathological staging, as per the ISGPS [17]. The total number of examined lymph nodes (ELNs), positive lymph nodes (PLNs), and lymph node ratio (LNR) are directly associated with the long-term survival of patients [5]. In this study, the average lymph node yield or ELN yield was 17.5 in SMD-PD and 11 in ST-PD. In ST-PD, mesentery along the first jejunal artery was not resected, but in SMD-PD (level 2), we removed mesentery along the first jejunal artery along with the specimen. This might be why the higher nodal yield in SMD-PD but with similar PLNs between both groups. The tumor-dependent factor, like LVI, was similar between both groups, but SMD-PD had fewer LVI-positive cases than ST-PD. The relevancy of these tumor-dependent factors will be evaluated during the follow-up of these cases. Our study has a few limitations. First, it is a retrospective study done on periampullary carcinoma. The mesopancreas dissection, especially level 3, provides an adequate resection margin in pancreatic ductal adenocarcinoma, frequently invading the superior mesenteric vein and artery. However, for periampullary carcinoma other than pancreatic ductal adenocarcinoma, the trend to invade SMV or SMA is not so common. Analysis of SMD as a whole could not be justified due to the lack of enough level 3 mesopancreas dissection in our study. The second drawback of this study is the change in the type of pancreaticojejunostomy and the omission of Braun's jejunojejunostomy in our last 12 cases. This change might alter or modify this study's intraoperative and early postoperative variables.

## Conclusions

SMD is safe and feasible for treating periampullary and pancreatic carcinoma and is particularly helpful in increasing lymph node yield. The long-term survival benefit of SMD in periampullary and pancreas cancer needs further prospective studies with longer follow-ups of patients.
